# Role of the Lifshitz topological transitions in the thermodynamic properties of graphene

**DOI:** 10.1039/d0ra04601a

**Published:** 2020-07-22

**Authors:** V. N. Davydov

**Affiliations:** M. V. Lomonosov Moscow State University Leninsky pr. 71, app. 121 117296 Moscow Russia vladimirdavydov@yahoo.com

## Abstract

The origin of the Lifshitz topological transition (LTT) and the 2D nature of the LTT in graphene has been established. The peculiarities of the Lifshitz topological transitions in graphene are described at the Brillouin zone centre Γ, the zone corners K in the vicinity of the Dirac points, and at the saddle point M. A general formulation of the thermodynamics at the LTT in graphene is given. The thermodynamic characteristics of graphene are investigated at the Lifshitz topological transitions. Anomalies are found in the electron specific heat *C*_e_, the electron thermal coefficient of pressure, and the coefficients of electron compressibility and thermal expansion in graphene at the LTT. All the thermodynamic parameters possess the strongest singularities in graphene at the LTT near the saddle points. This opens exciting opportunities for inducing and exploring the Lifshitz topological transitions in graphene.

## Abstract 2

The origin of the Lifshitz topological transition (LTT) and the 2D nature of the LTT in graphene has been established.

## Graphical abstract

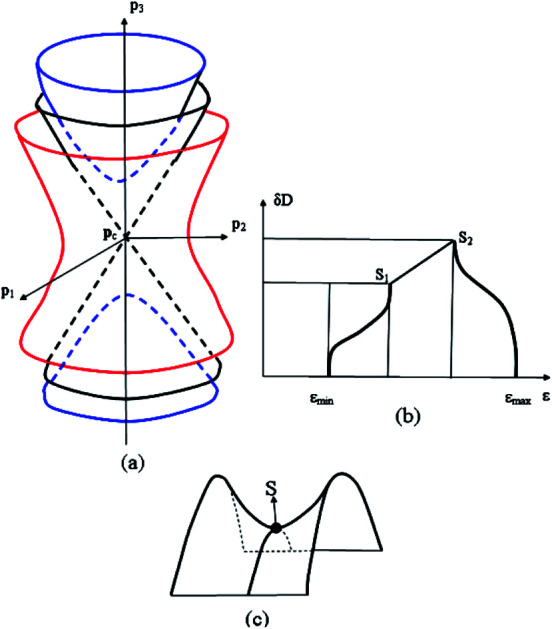

## 1 Introduction

Anomalies in the thermodynamic quantities of metals due to a change in the topology of the Fermi surface *ε*(**p**) = *ε*_F_ are customarily called the Lifshitz topological transition (LTT) (I. M. Lifshitz;^1^ see also ref. 2, 3 and 4). It is understood that a change in the topology of the Fermi boundary surface is due to the continuous variation of some parameter (*e.g.*, pressure), as a result of which the difference *z* = *ε*_F_ − *ε*_c_ (where *ε*_F_ is the Fermi energy and *ε*_c_ is the critical energy at which the topology of the constant energy surface changes) passes through zero continuously. This leads to a change in the connectivity of the Fermi surface (the appearance of a new cavity, the rupture of a connecting neck, *etc.*), and at absolute zero temperature *T* = 0 K, the grand thermodynamic potential Ω (often called the Landau free energy or Landau potential^5^) acquires an irregular correction:

1.1 *δΩ* = −*α*|*z*|^5/2^.

It can be seen that the third derivatives of the thermodynamic potential become infinite at the point *z* = *μ* − *ε*_c_ = 0 (the chemical potential *μ* is equal to the Fermi energy *ε*_F_ at *T* = 0); therefore, this change in the topology of the Fermi surface is also called the 2½-order phase transition (FT2½) according to the Ehrenfest terminology.^6,7^

The anomalies arising in the LTT manifest themselves at *T* ≪ *ε*_F_ not only in the thermodynamic characteristics of metals but also in other characteristics (*e.g.*, magnetic-field dependence of the electrical resistance,^8^ sound absorption^9–15^). It has been shown in ref. 16 that the thermoelectric power has the square root divergency at the LTT. The discovery of graphene^17^ gave new inspiration to investigations of the Lifshitz transition properties. To date, numerous papers have been devoted to the Lifshitz transitions in bilayer (BLG) and multilayer (MLG) graphene.^18–30^ The results of these studies indicate that the effects of the LTT are appreciable and can be used to observe the 2½-order transitions as well as to investigate the degree of smearing of the Fermi surface in metals. In ref. 31, experimental evidence was obtained of the Lifshitz transition in the thermoelectric power of ultrahigh mobility bilayer graphene. Resolving low-energy features in the density of states (DOS) holds the key to understanding a wide variety of rich and novel phenomena in graphene based on 2D heterostructures. The Lifshitz transition in bilayer graphene (BLG) arising from trigonal warping has been established in^31^ theoretically and experimentally.

The 21st century has brought many new results related to graphene thermodynamics.^32–45^ Apparently, the thermodynamics of graphene has been characterized from many points of view; however, the role of the Lifshitz topological transitions in the thermodynamic properties of graphene has not been studied. This paper aims to close this gap. Because graphene is the first real two-dimensional solid, a general formulation of the thermodynamics at the LTT in graphene is given. The unusual thermodynamic properties of graphene stem from its 2D nature, forming a rich playground for new discoveries of heat flow physics and potentially leading to novel thermal management applications.

This paper is arranged as follows. In section 2, the origin of the Lifshitz topological transition and the 2D nature of the LTT in graphene are considered. Peculiarities of the Lifshitz topological transitions in graphene are then investigated in section 3. The thermodynamics of graphene at the Lifshitz topological transitions is proposed in section 4. Finally, conclusions are drawn in section 5.

## 2 Origin of the Lifshitz topological transitions and connection of the two-dimensional nature of graphene with the van Hove singularities

It is known [see ref. 1, 2, and 3] that at the LTT, a new cavity of the electronic isoenergetic surface appears (or disappears) at the critical energy *ε*_c_ in the critical point of the momentum space **p** = **p**_c_, where the electron energy as a function of quasimomentum *ε* = *ε*(**p**) has a minimum *ε*_min_ or maximum *ε*_max_. In this case, the isoenergetic surfaces in the vicinity of **p** = **p**_c_ are well described by the ellipsoid equations:2.1
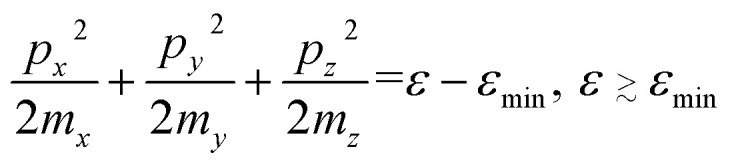
2.2
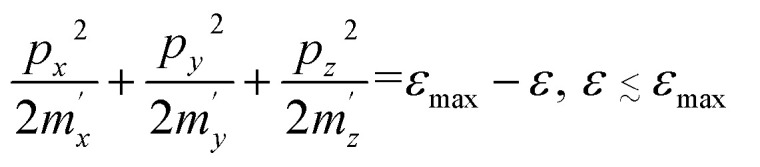
where *m*_*x*_, *m*_*y*_, and *m*_*z*_ are the main values of the effective mass tensor in the vicinity of *ε*_min_ (see ref. 46 and 47);
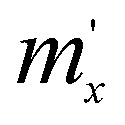
,
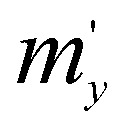
, and 
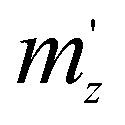
 are the main values of the effective mass tensor in the vicinity of *ε*_max_.^45^

**Fig. 1:**
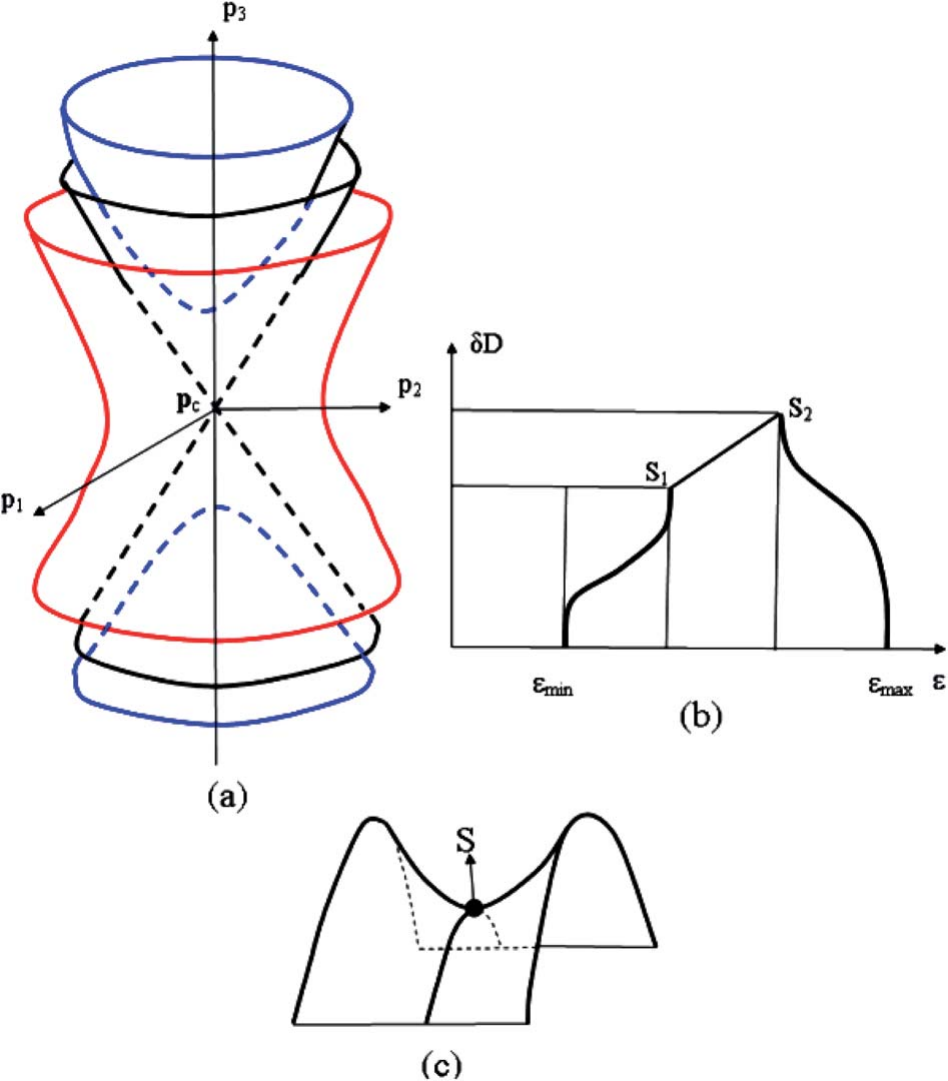
The cone point at **p** = **p**_c_ (a), the saddle point (c), and the van Hove singularities of the state density *δD*(*ε*) (b). S, S_1_, and S_2_ are the saddle points.

At the neck rupture, the boundary isoenergetic surface *ε*(**p**) = *ε*_c_ contains the peculiar point of another type, named the cone point, at **p** = **p**_c_. In this case, the isoenergetic surface in the vicinity of the cone point **p** = **p**_c_ is described by the hyperboloid of two sheets at energy *ε* < *ε*_c_ and the hyperboloid of one sheet at energy *ε* > *ε*_c_ (Fig. 1a):2.3
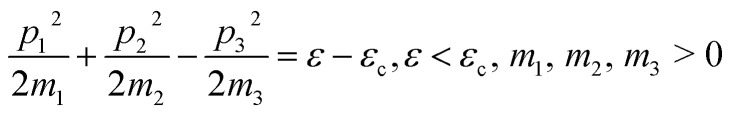
2.4
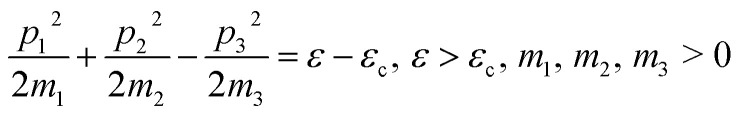


At energies close to *ε*_c_, one can express the electron density of states (DOS) as

2.5 *D*(*ε*) = *D*_0_(*ε*) + *δD*(*ε*)

where *D*_0_(*ε*) is the regular smooth function of the energy, and *δD*(*ε*) depends on the type of LTT. The latter is computed by the relation (per spin direction and per unit volume in 3D-space, or per unit area in 2D-space):2.6
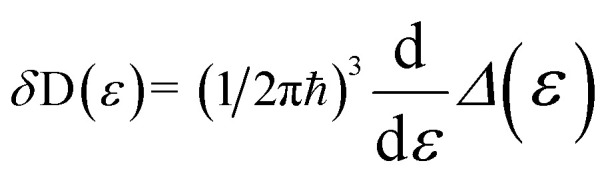
where *ħ* is the reduced Planck–Dirac constant, and *Δ*(*ε*) is volume of the ellipsoid (2.1) or (2.2). At the neck rupture (Fig. 1a), *Δ*(*ε*) is the change of the volume enclosed by the plane *p*_3_ = *p*_0_ and the hyperboloid of one (2.3) or two (2.4) sheets (Fig. 1a) in the disruption of the isoenergetic surface neck in the vicinity of the cone point **p** = **p**_c_.

One can join eqn (2.1)–(2.4) into a single equation:2.7
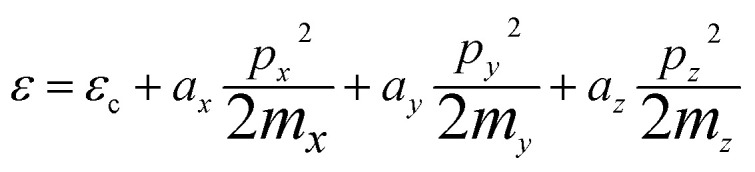
where *m*_*i*_ (*i* = *x*, *y*, *z*) are the positive values of the effective mass tensor, *m*_*i*_ ≠0, and integers *a*_*j*_ (*j* = *x*, *y*, *z*) are equal to ±1.

Four types of singularities exist of the density of the electron states in three-dimensional space, and the singularity type depends on the signs of the coefficients *a*_*j*_.

The point M_0_ (min) corresponds to the case where all three coefficients *a*_*j*_ = +1. This point corresponds to the minimum in the energy spectrum.

The point M_1_ (saddle) corresponds to the case where two coefficients *a*_*j*_ are positive and the third one is negative. This is the saddle point (Fig. 1c).

The point M_2_ (saddle) is the case where two coefficients *a*_*j*_ are negative and a third one is positive. This is the second saddle point (Fig. 1b).

The point M_3_ (max) corresponds to the case where all three coefficients *a*_*j*_ = −1. This point corresponds to the maximum in the energy spectrum.

There are three types of singularities of the density of the electron states in two-dimensional space, and the singularity type depends on the signs of the coefficients *a*_*j*_ (*j* = *x*, *y*).

The point P_0_ (min) corresponds to the case where both coefficients *a*_*j*_ = +1. This point corresponds to the minimum in the energy spectrum.

The point P_1_ (saddle) corresponds to the case where one coefficient *a*_*j*_ is positive and another one is negative. This is the saddle point.

The point P_2_ (max) corresponds to the case where both coefficients *a*_*j*_ = −1. This point corresponds to the maximum in the energy spectrum.

There are two types of singularities of the density of the electron states in one-dimensional space:

• The point Q_0_ (min) corresponds to the case where coefficient *a* = +1. This point corresponds to the minimum in the energy spectrum.

• The point Q_1_ (max) corresponds to the case where coefficient *a* = −1. This point corresponds to the maximum in the energy spectrum.

The analytical behavior of the density of the electron states at LTT is given in Table 1. The results are computed from formulae (2.1)–(2.7); see also ref. 1, 2, and 4.

**Table 1:** The analytical behavior of the density of the electron states at the Lifshitz topological transitions

Dimensionality	Type of singularity	Density of the electron states at *ε*_c_		
		*ε* < *ε*_c_	*ε* > *ε*_c_	Coefficients *C*_*i*_
3D	M_0_ (min)	0	*C*_1_ (*ε* − ε_c_)^1/2^	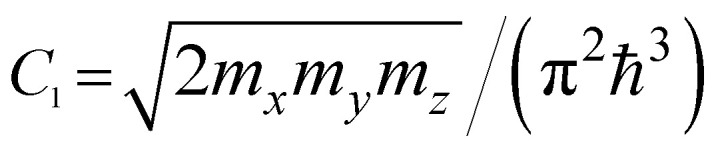
	M_1_ (saddle)	*C*_2_ − *C*_3_(*ε*_c_ − *ε*)^1/2^	*C*_2_	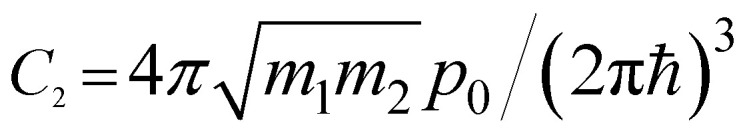
	M_2_ (saddle)	−*C*_2_	*C*_2_ − *C*_3_(*ε* − ε_c_)^1/2^	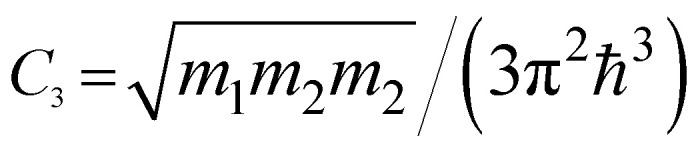
	M_3_ (max)	*C*_4_(*ε*_c_ − *ε*)^1/2^	0	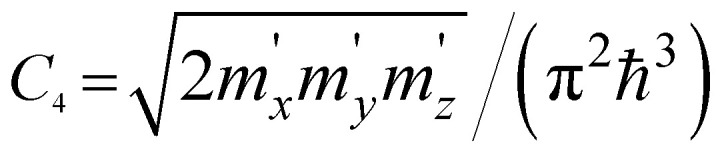
2D	P_0_ (min)	0	*C*_5_	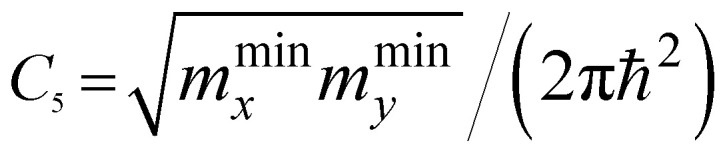
	P_1_ (saddle)	−*C*_6_ ln|1 − *ε*/*ε*_c_|	*C*_6_ ln| *ε*/*ε*_c_ − 1|	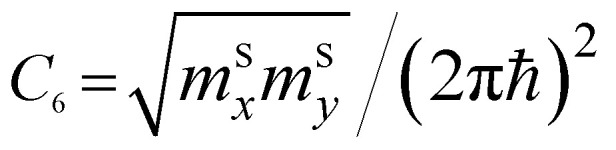
	P_2_ (max)	*C*_7_	0	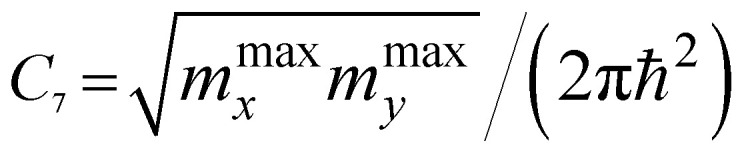
1D	Q_0_ (min)	0	*C*_8_ (*ε* − ε_c_)^−1/2^	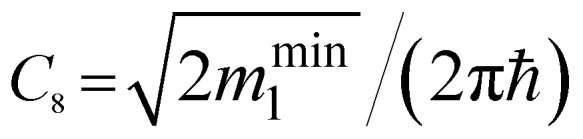
	Q_1_ (max)	−*C*_9_(*ε*_c_ − *ε*)^−1/2^	0	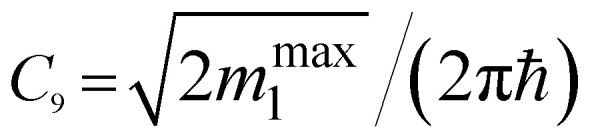

We can conclude based on the square root peculiarities of the density of the electron states in three dimensions (Table 1) at *ε*_c_ that nature of the Lifshitz topological transition stems from the van Hove singularities (VHS) of the state density *δD*(*ε*).^48–52^ In this connection, the van Hove topological theorem^48^ states that the spectrum must contain at least one of the saddle-points S_1_ and S_2_ (Fig. 1b), and the slope of *D*(*ε*) must tend to −∞ on the upper end.

The two-dimensional nature of graphene should exhibit special types of van Hove singularities and LTTs. This statement is illustrated by the general argument that in two dimensions, the saddle-points produce logarithmic singularities (Table 1), and the spectrum extrema produce finite discontinuities of the electron density of states [also see ref. 49, 53, and 54]. This is valid for the elementary excitations of the quasiparticles with values *m*_*i*_ ≠ 0 of the effective mass tensor; however, it is not applied to the massless Dirac fermions in graphene. If the logarithmic singularity is a general property of 2D electronic systems in the saddle points, this statement should be valid for 2D graphene. However, the latter is in contradiction with 
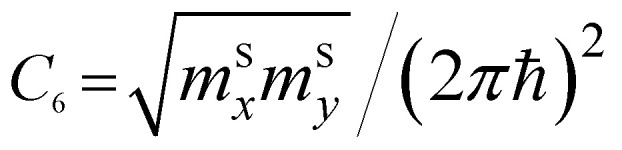
 because the value of *C*_6_ must be zero for the massless fermions in graphene. Here, the discrepancy arises because the logarithmic singularity vanishes. We will resolve this contradiction in section 3.

Therefore, the Lifshitz topological transitions in graphene require special investigation. The latter was also confirmed by recent work.^55^ It has been demonstrated in^55^ theoretically that the characteristic feature of a 2D system undergoing N consequent Lifshitz topological transitions is the occurrence of spikes of entropy per particle *s* of a magnitude ± ln 2/(*J* − 1/2) with 2 ≤ *J* ≤ *N* at low temperatures.

## 3 Peculiarities of the Lifshitz topological transitions in graphene

**Fig. 2:**
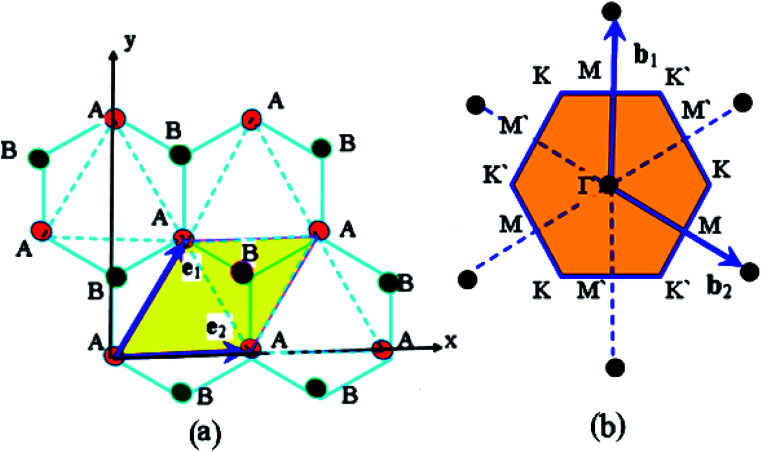
(Reproduced and adapted with permission from ref. 56). The crystal structure of the single graphite layer (a). The primitive translation vectors **e**_1_ and **e**_2_ form the rhombic unit cell, and the basis consists of two C atoms, shown as A and B. The Bravais lattice (consider, *e.g.*, the lattice formed by the A atoms) is triangular with a Bravais lattice spacing of 
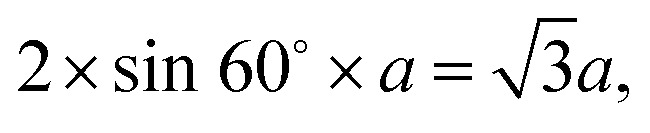
 where *a* is the spacing between neighboring atoms. The graphene reciprocal lattice and the first Brillouin zone (b).

A single layer of graphene consists of carbon atoms in the form of a honeycomb lattice (Fig. 2). The primitive translation vectors **e**_1_ and **e**_2_ form the rhombic unit cell. The hexagonal lattice consists of two trigonal sublattices AAA and BBB.^56^ There are four valence electrons (two 2s and two 2p electrons). Three of those participate in the chemical bonding and thus are in bands well below the Fermi energy. We therefore consider the bands formed by the one remaining electron. We assume a tight-binding model in which the electron hops between neighboring atoms. We denote the spacing between neighbouring atoms by a.

From Fig. 2a, we see that two basis vectors of the Bravais lattice are3.1
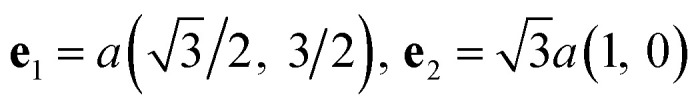


The reciprocal Bravais lattices, **b**_1_ and **b**_2_, are defined such that

3.2 **b**_*i*_·**e**_*j*_ = 2π*δ*_*ij*_

The result is3.3
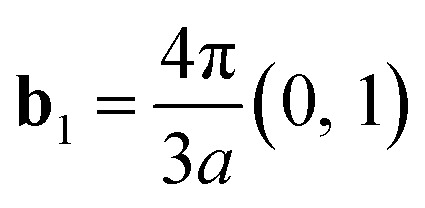
3.4
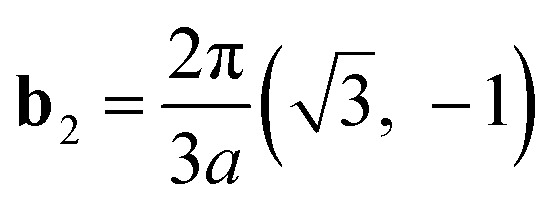


These basis vectors are of equal length and at 120°; therefore, the reciprocal lattice is a hexagonal lattice (Fig. 2b). The first Brillouin zone is shown in Fig. 2b. The first Brillouin zone is a regular hexagon, whose most characteristic points are its centre Γ, the inequivalent corners K and K′, and the centres of the lateral edges M and M'. The distance ΓM is 
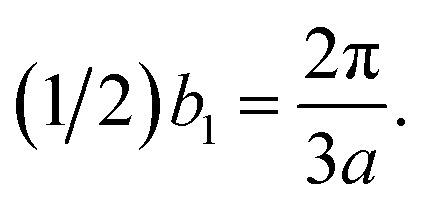
 The distance ΓΚ is 
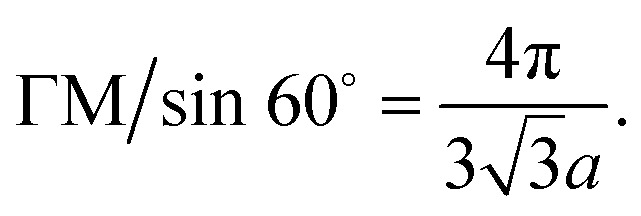


Consider a state with amplitude *Ψ*_**R**_ for the electron to be at the site labeled by **R**. We look for the wave functions with amplitudes which vary, such as e^**ik·r**^. There will be different amplitudes *Ψ*_A_ and *Ψ*_B_ on sublattices A and B, so *Ψ*_**R**_ = *Ψ*_A_e^**ik·r**^ (**R** ∈ A), and *Ψ*_**R**_ = *Ψ*_B_e^**ik·r**^ (**R** ∈ B). An electron at site **R** can hop to any of the neighboring sites. An atom on sublattice AAA has neighboring atoms (Fig. 2a), all on sublattice BBB, at displacements (0, *a*), 
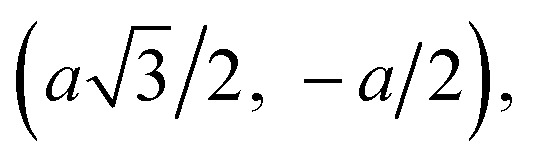
 and 
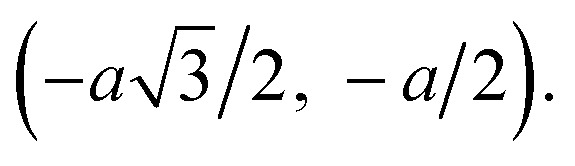
 An atom on sublattice BBB has three neighbors on sublattice AAA at displacements (0, −*a*), 
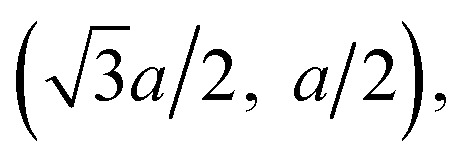
 and 
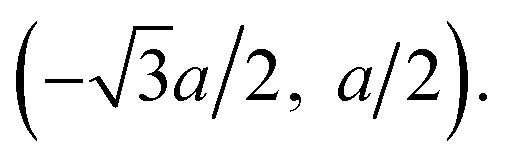
 In the nearest-neighbour approximation, there are no hopping processes within the sublattices AAA and BBB; hopping only occurs between them. Hence, the eigenvalue *ε* and the amplitudes *Ψ*_A_ and *Ψ*_B_ are determined for each wavevector **k** from two equations:3.5
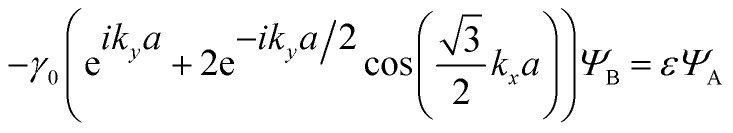
3.6
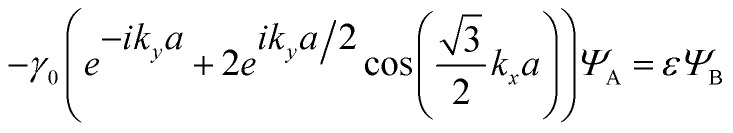
where *γ*_0_ is the hopping parameter between nearest neighbors.

We write the Hamiltonian in the tight-binding approximation for the wave vector **k** = **p**/*ħ* (where **p** is the electron quasimomentum), taking into account eqn (3.5) and (3.6), as3.7
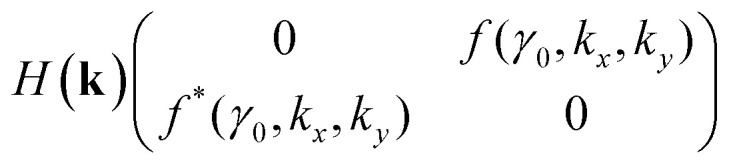
where3.8
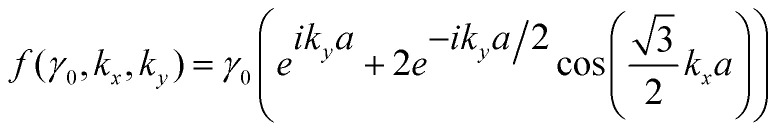


The electron energy eigenvalues of the Hamiltonian (3.7) are given by3.9
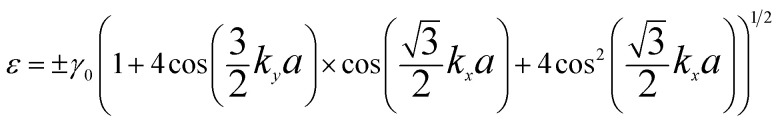


In expression (3.9), the plus and minus signs correspond to the conduction and valence bands, respectively.

To explore the possible Lifshitz topological transitions, consider the structure of the isoenergetic lines in the (*k*_*x*_, *k*_*y*_) plane for different positions of the wave vector **k**.

(i) The maximum and minimum energies are *ε*_c_ = ±3*γ*_0_, and they occur at **k** = 0, *i.e.* in the centre Γ (Fig. 2b) of the first Brillouin zone. The value of *ε*_c_ = 3*γ*_0_ corresponds to the maximum of energy in the conduction band (the point P_2_ (max) in Table 1). The value of *ε*_c_ = −3*γ*_0_ corresponds to the minimum of energy in the valence band (the point P_o_ (min) in Table 1). Taking into account |*k*_*y*_*a*|, |*k*_*x*_*a*| ≪ 1, from (3.9), we have accuracy of order (*k*_*y*_*a*)^2^, (*k*_*x*_*a*)^2^:3.10
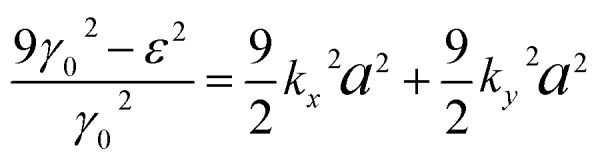


Thus, the isoenergetic lines are circles in the vicinity of the extrema of energy at **k** = 0. It follows from (3.10) that these circles are described by the following equation in **p**-space:3.11
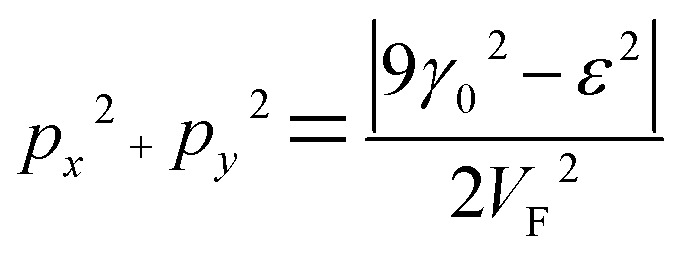
where3.12
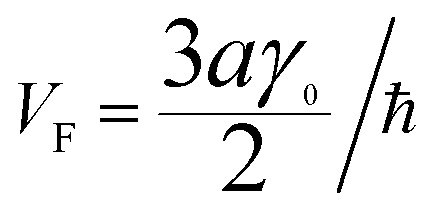


**Fig. 3:**
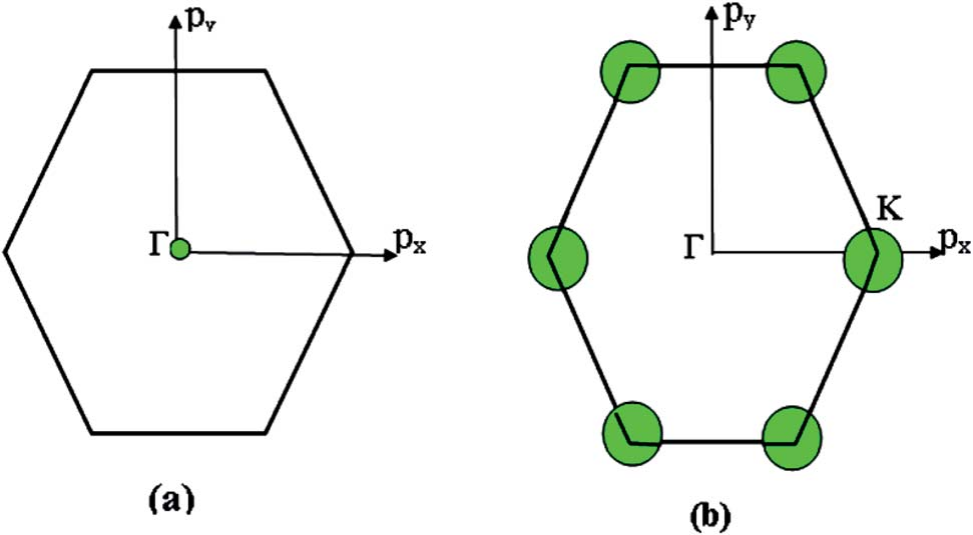
(a) Disappearance of the cavity of the isoenergetic lines in the Brillouin zone centre Γ at the critical value of energy *ε*_c_ = 3*γ*_0_ in the conduction band. Appearance of the cavity of the isoenergetic lines in the Brillouin zone centre Γ at the critical value of energy *ε*_c_ = −3*γ*_0_ in the valence band. (b) Appearance of new cavities in the conduction band in the corners K of the Brillouin zone at the critical value of energy *ε*_c_ = 0 in the extended zones scheme. Disappearance of new cavities in the valence band in the corners K of the Brillouin zone at the critical value of energy *ε*_c_ = 0 in the extended zones scheme.

Consequently, there exist LTT at critical values of energy *ε*_c_ = ±3*γ*_0_, where the cavity (3.11) of the isoenergetic lines disappears (Fig. 3a) at *ε*_c_ = +3*γ*_0_ in the conduction band or appears at *ε*_c_ = −3*γ*_0_ in the valence band.

The number of the electron states inside the two-dimensional cavity (per spin direction and per unit area) is equal to

3.13 *δN*(*ε*) = (1/2π*ħ*)^2^*Δ*(*ε*)

where *Δ*(*ε*) is the area of the cavity in the momentum space.

We obtain from (3.11) and (3.13)3.14
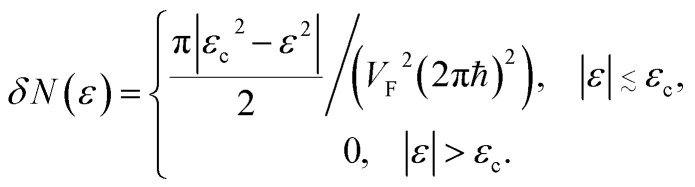
where *ε*_c_ = 3*γ*_0_.

One can represent the change of the density of states *δD*(*ε*) due to LTT as3.15
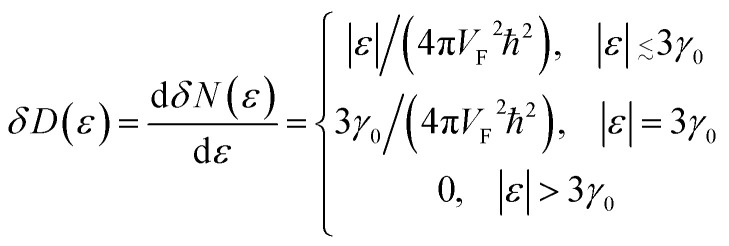


The discontinuity at the end points *ε* = ±3*γ*_0_ comes from a maximum or minimum of the dispersion relation in two dimensions. The analytic expression without derivation for the total DOS in graphene for model (3.7) was provided by Hobson and Nierenberg in 1953.^53^ In a recent paper,^54^ the expression for this model was derived for the total DOS (per unit hexagonal cell of area 
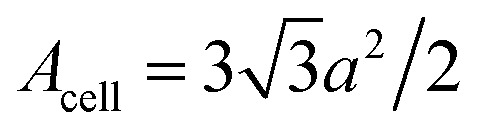
 (Fig. 2a) and one spin component), valid for region 0 < |*ε*| < 3*γ*_0_:3.16
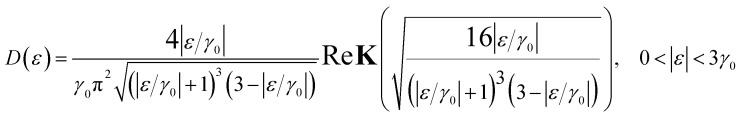
where **K**(*ξ*) is the complete elliptic integral of the first kind, *i.e.*3.17
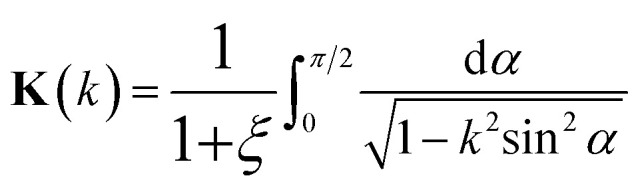
3.18
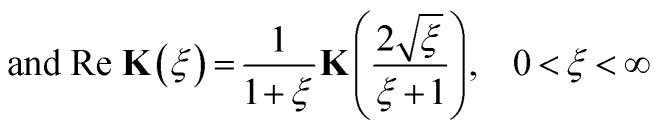


Expanding of (3.16) near the point Γ in the small region |*ε*| ≲ 3*γ*_0_ in the vicinity of the LTT points |*ε*_c_| = 3*γ*_0_, one obtains (per spin direction and per unit area):3.19
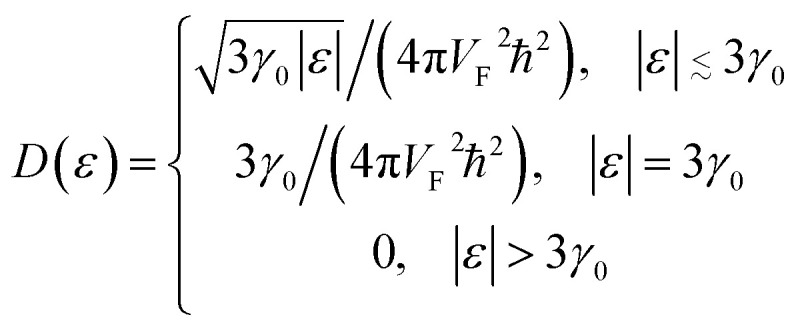


The result (3.15) for *δD* (*ε*) coincides with the total *D*(*ε*) given by (3.19) in the region |*ε*| ≲ 3*γ*_0_ in the vicinity of the LTT points |*ε*_c_| = 3*γ*_0_, where 
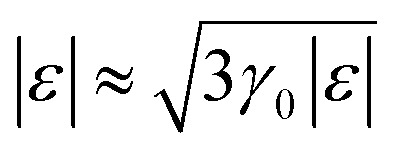
at |*ε*| ≲ 3*γ*_0_.

As follows from (3.19), for graphene in the vicinity of the point Γ, the coefficients *C*_5_ and *C*_7_ in Table 1 should be changed as3.20
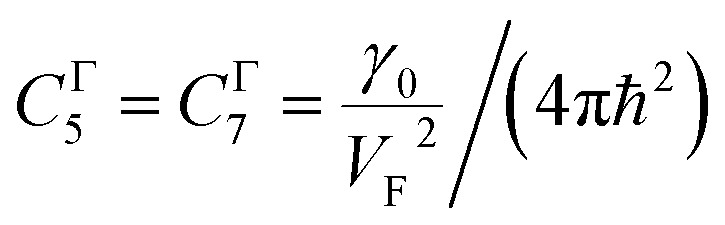


Hence, in 2D graphene, the mass-factors of coefficients *C*_5_ and *C*_7_ in Table 1 acquire the substitutions of *γ*_0_/*V*_F_^2^ instead of 
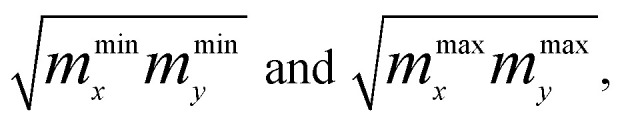
 respectively, and this substitution can be treated as the fermion effective mass in the vicinity of the point Γ:3.21
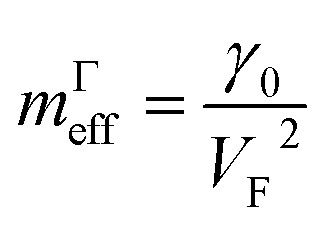
(ii) Now, we compute the dispersion relation in the vicinity of the zone corners K(K′), where the energy tends to zero. We write

3.22 **k** = **K** + **κ**

where **K** is the wavevector at the zone corner, 
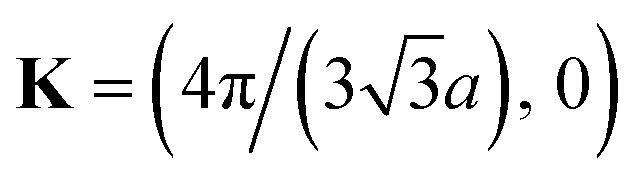
 for example, and we will assume that **κ** is small.

To the lowest order in *κ* we have, from (3.8) and (3.22),3.23
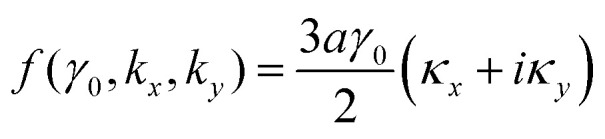


Substitution of (3.23) into Hamiltonian (3.7) gives the following equation for the isoenergetic lines in vicinity of the point K (the corner of the first Brillouin zone):3.24
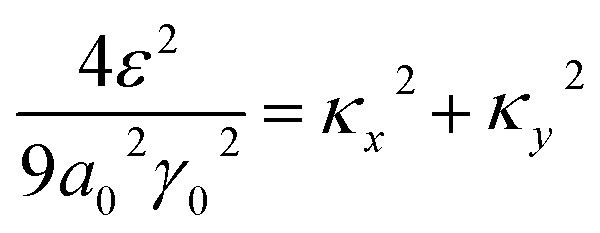


Consequently, the isoenergetic lines are circles in the vicinity of the minimum of energy *ε*_c_ = 0 (for the conduction band) or in the vicinity of the maximum of energy *ε*_c_ = 0 (for the valence band) at the corner of the zone. It follows from (3.24) that these circles are described by the following equation in **p**-space (Fig. 3b):3.25
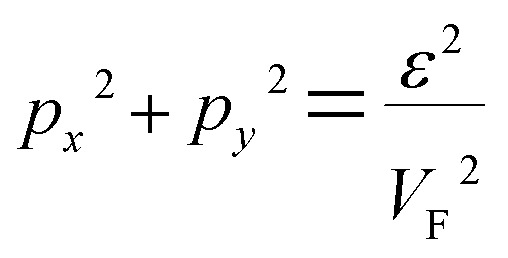


The value of *ε*_c_ = 0 corresponds to the minimum of energy in the conduction band (the point P_0_ (min) in Table 1). The value of *ε*_c_ = 0 corresponds to the maximum of energy in the valence band (the point P_2_ (max) in Table 1). Eqn (3.25) describes the linear dispersion of the massless Dirac fermions in the vicinity of the corners K(K′) of the first Brillouin zone:

3.26 *ε* = ±V_F_p

Thus, the LTT exists at the critical value of energy *ε*_c_ = 0, where the new cavity (3.25) appears (Fig. 3b) in the conduction band or disappears in the valence band (Fig. 3b). Hence, there are six pockets of low energy excitations (Fig. 3b), one for each of the two inequivalent points K and K′ on the Brillouin zone boundary.

The area of the cavity inside the circle (3.25) is equal to.3.27
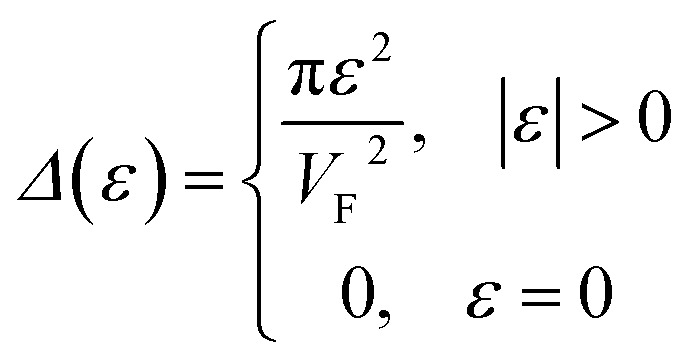


Using (3.13) and (3.27) to calculate the number of the electron states inside the two-dimensional cavity (3.25), one obtains (per spin direction and per unit area):

3.28 *δN*(*ε*) =π*ε*^2^/[*V*_F_^2^(2π*ħ*)^2^]

Computing DOS from (3.28), one has3.29
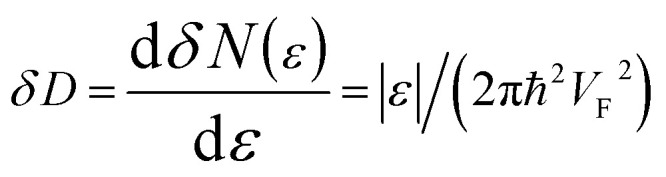


Expanding the expression (3.16) at *ε* = *ε*_c_ = 0, we obtain the same result for the total DOS (per spin direction and per unit area) in the vicinity of the zone corners K(K′):

3.30 *D*(*ε*) =|*ε*|/(2π*ħ*^2^*V*_F_^2^)

Therefore, in the points K(K′), we must make the following substitutions for the coefficients *C*_6_ and *C*_7_ for graphene in Table 1:3.31
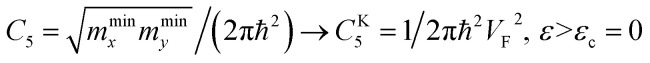
3.32
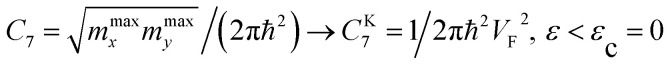
(iii) Let us compute the dispersion relation in the vicinity of the zone edges, *e.g.* near the middle M of the boundary of the first Brillouin zone. We write

3.33 **k** = **K** + **q**

where **K** is the wavevector at point M of the zone boundary (Fig. 2b). The distance ΓM to the center of the edge of the zone is
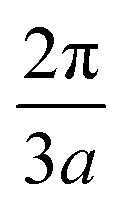
. Therefore:3.34
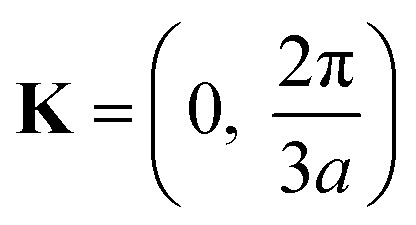
and we will assume that **q** is small.

Taking into account |*q*_*y*_*a*|, |*q*_*x*_*a*| ≪ 1, we have from (3.9), (3.33) and (3.34) to accuracy of order (*q*_*y*_*a*), (*q*_*x*_*a*)3.35
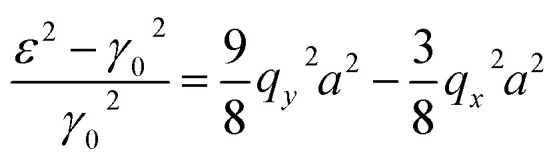


**Fig. 4:**
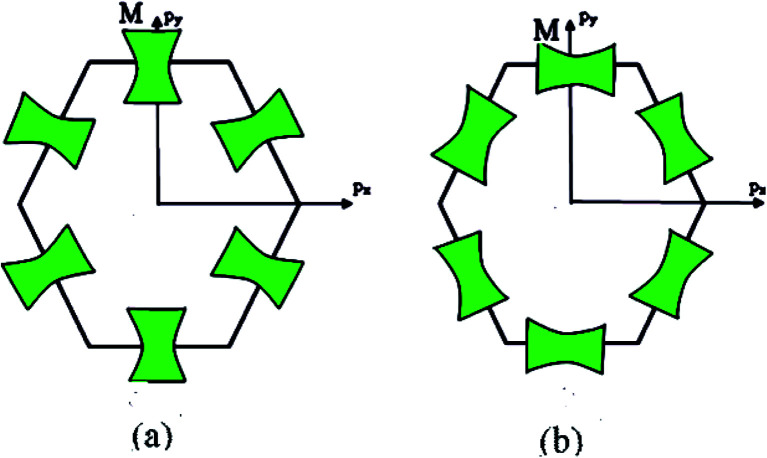
The topology changes of the isoenergetic lines in the vicinity of the saddle points M in the extended zones scheme: (a) at the critical value of energy |*ε*| < *γ*_0_; (b) at the critical value of energy |*ε*| > *γ*_0_.

Therefore, the isoenergetic lines are hyperboles in the vicinity of point M (the middle of the edge of the first Brillouin zone). One can say that middles of the edges of the first Brillouin zone are saddle points. We can consider the corresponding points similar to M (the middles of the edges of the first Brillouin zone) (Fig. 2b) as the saddle points or the “cone points” in two dimensions (points P_1_ (saddle) in Table 1). It follows from (3.35) that the isoenergetic lines are described by the following equations in **p**-space in the vicinity of these saddle points (Fig. 4):3.36
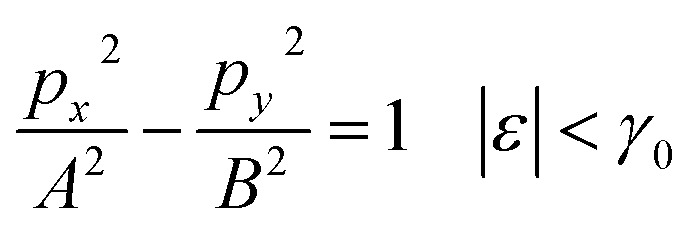
3.37
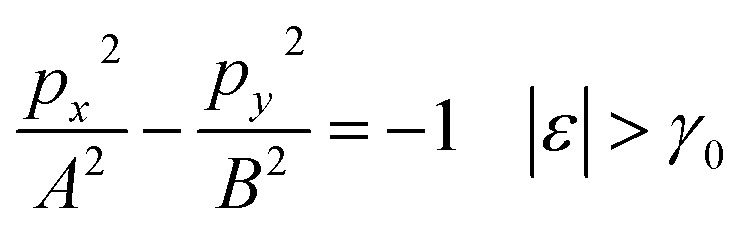
where3.38
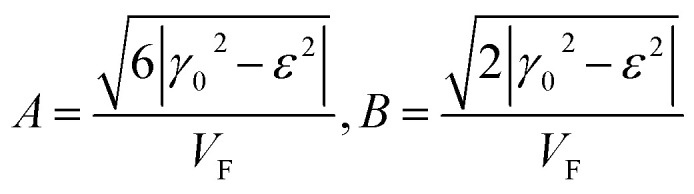


The Lifshitz topological transition is realized in the saddle point M by the variation of energy from *ε* < *ε*_c_ to *ε* > *ε*_c_, and *ε*_c_ = *γ*_0_. In this transition, the isoenergetic lines in the vicinity of point M change from hyperbole (3.36) to hyperbole (3.37). Calculating the area *Δ*(*ε*) enclosed by hyperbole (3.36) or (3.37) and the corresponding boundary of the first Brillouin zone *p*_*y*_ = *ħ*/*a*, or *p*_*x*_ = *ħ*/*a*, one obtains:3.39
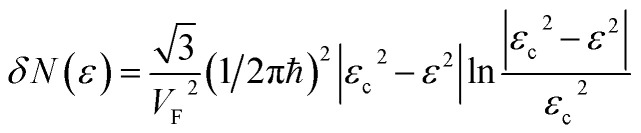
where *ε*_c_ = *γ*_0_.

Now, we are able to resolve the contradiction with the coefficient 
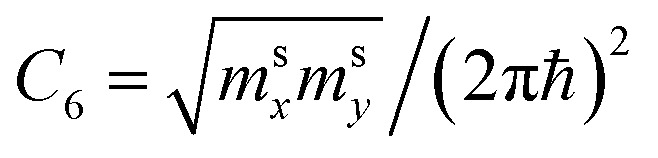
 used in Table 1. As follows from (3.39) and (3.13), the DOS change due to the LTT in the saddle point M (at energies close to *ε*_c_) is equal to3.40
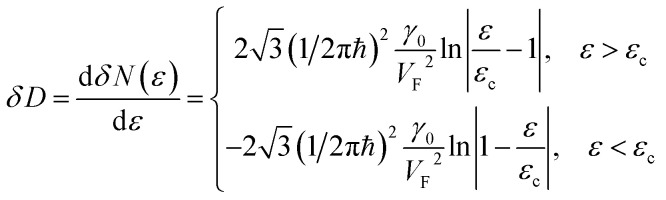


Also, the expansion of (3.16) yields the same VHS for the total DOS (per spin direction and per unit area) in the vicinity of the saddle point M, if |*ε*| → *ε*_c_ = *γ*_0_:3.41
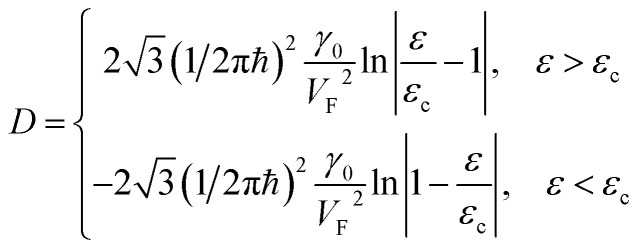


Therefore, in the saddle point M, the coefficient *C*_6_ for graphene in Table 1 should be changed to3.42
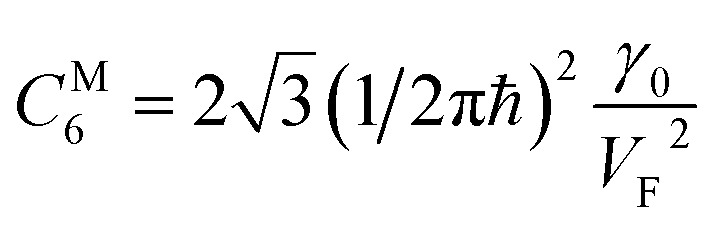


Thereby, in 2D graphene, the mass-factor *C*_6_ in Table 1 acquires the substitution of *γ*_0_/*V*_F_^2^ instead of
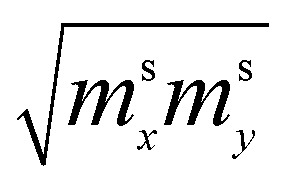
, and this substitution can be treated as the graphene effective mass in the vicinity of the saddle point M:3.43
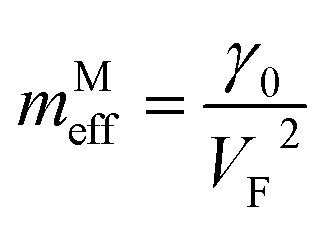


It follows from (3.21) and (3.43) that the fermions are slowing down in the vicinities of the Γ and M points, becoming not massless but massive. A similar phenomenon was found in observations of Dirac node formation and mass acquisition in a topological crystalline insulator.^57^ Estimation of the values of *m*^Γ^_eff_and *m*^M^_eff_yields (at *γ*_0_ = 4 eV, and *V*_F_= 10^8^ cm s^−1^ (ref. 56)):

3.44 *m*^Γ^_eff_ ≈ 0.1*m*_e_

3.45 *m*^M^_eff_ ≈ 0.1*m*_e_

where *m*_e_ is the free electron rest mass.

## 4 Contribution of the Lifshitz topological transitions to the thermodynamic properties of graphene

### 4.1. The thermodynamic potentials of graphene near the Lifshitz topological transition

Consider the thermodynamic properties of graphene near the electronic transition caused by the topology change of the Fermi lines in graphene. If the chemical potential *μ* is close to a value of the critical energy *ε*_c_, the grand thermodynamic potential *Ω* (ref. 5) has the following expression:^2^

4.1 *Ω*(*μ*, *T*) = *Ω*_0_ + *δΩ*

where4.2
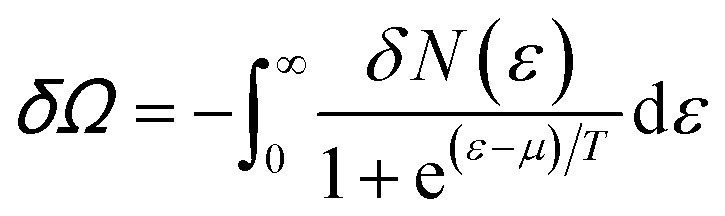
and the temperature *T* is measured in ergs.

Introducing the Lifshitz parameter *z* = *μ* − *ε*_c_ in the case of the hyperbolic changes of the Fermi lines at the saddle point M, we obtain from (3.39) and (4.2), if *T* → 0:4.3
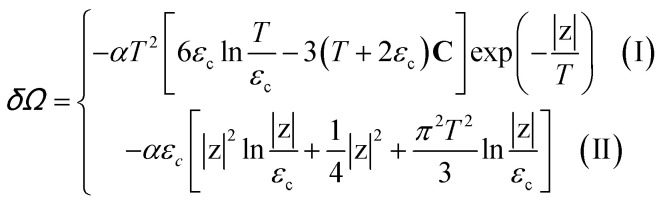
where 
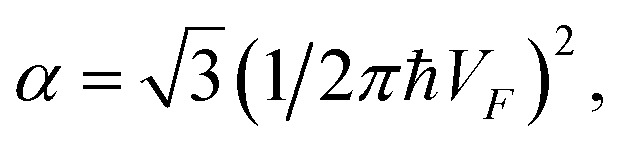
*z* = *μ* − *ε*_c_, *ε*_c_ = *γ*_0_, and **C** is the Euler constant.

Transition from region I into region II corresponds to the appearance of a new cavity of the isoenergetic line ε(**p**) = *μ*, or to a decrease of the line connectivity.

Both formulae (4.3) are valid at *T* ≪ |*z*|. At absolute zero temperature, one has4.4
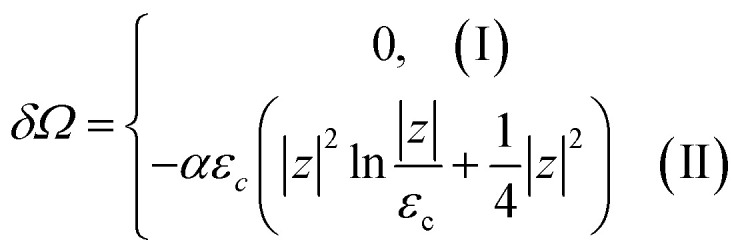


From comparison of (4.4) and (1.1), one can see the essential difference of *δΩ* given by (4.4) for the LTT in graphene and the LTT in three dimensions (1.1). The second derivative of *δΩ* diverges at the point *z* = 0 as ln(|*z*|/*ε*_c)_in graphene, while only the third derivative of *δΩ* diverges at the point *z* = 0 as z^−1/2^ in three dimensions. As a result, completely different anomalies will be obtained for the thermodynamic parameters at the Lifshitz topological transitions in graphene.

When the electronic cavity disappears in the Brillouin zone centre Γ, one computes from (3.14) and (4.2) at *T* → 0:4.5
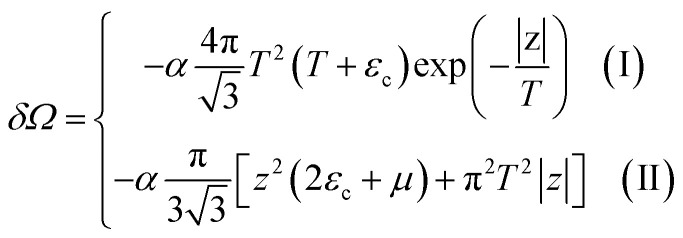
where *z* = *ε*_c_ − *μ*, *ε*_c_= 3*γ*_0_.

Considering the appearance of new cavities in the Brillouin zone corners K, we obtain from (3.28) and (4.2), if *T* → 0:4.6
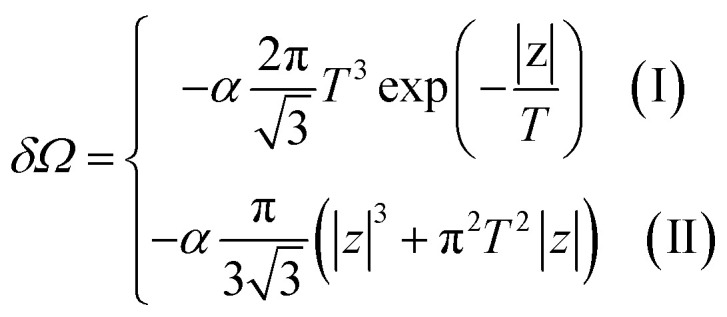
where *z* = *μ* − *ε*_c_, *ε*_c_= 0.

Because the number of electrons in the conduction band is permanent (at least in vicinity of the point *μ* = *ε*_c_), it is convenient to use the Helmholtz free energy potential *F* (*T*, *S*, *N*)^2^ instead of the grand thermodynamic potential *Ω* (*T*, *S*, *μ*). We assume that area *S* is the parameter connected with applied pressure, tensile stress, or other mechanical action. The critical energy is the function of the area, *i.e. ε*_c_ = *ε*_c_(*S*), and the chemical potential *μ* is also a function of *S* because of constancy of the carriers:

4.7 *N*(*μ*, *S*) = *N*

If *S*_c_ is the area at which the topology of the Fermi lines changes:

4.8 μ(*S*_c_) = *ε*_c_(*S*_c_)

According to (4.7) and (4.8), the value of |*z*| = |*μ* − *ε*_c_| can be expressed *via* |*S* − *S*_c_|:

4.9 |*z*| = *η*|*S* − *S*_c_|

where *η* is independent of *S*, and *η* = *η*(*μ*).

The Helmholtz free energy potential *F* can be written in the form

4.10 *F* = *F*_0_ + *δF*

where *F*_0_ is the smooth part of the free energy.

One can readily be convinced that *δF* is quantitatively equal to the irregular contribution *δΩ* expressed in the variables *S* and *T* according to the Landau theorem^5^ about the small variations of the thermodynamic potentials due to small changes of some parameters of the solid state:

4.11 (*δΩ*)_*T*,*S*,*μ*_ = (*δF*)_*T*,*S*,*N*_

One can see that variations of *δΩ* given by the relations (4.3), (4.5), and (4.6) are small at *T* ≪ *z* in the vicinity of the Lifshitz electronic transition, where *z* → 0.

Thus, *δF* is given by eqn (4.3), (4.5), and (4.7), where one must set |*z*| = *η*|*S* − *S*_c_|.

In the case of the hyperbolic changes of the Fermi lines at the saddle point M, we obtain from (4.3) and (4.9), if *T* → 0:4.12
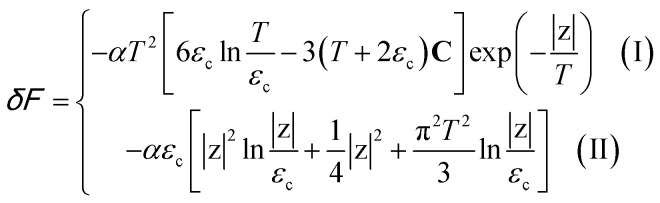
where |*z*| = *η*_*γ*_0__|*S* − *S*_c_|, *η*_*γ*_0__ = *η*(*μ*→*γ*_0_).

When the electronic cavity disappears in the Brillouin zone centre, one obtains from (4.5) and (4.9) at *T* → 0:4.13
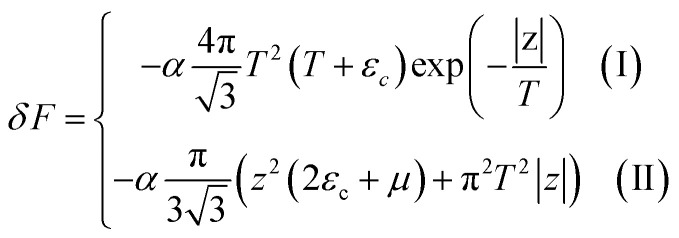
where |*z*| = *η*_3*γ*_0__|*S* − *S*_c_|, *η*_3*γ*_0__ = *η*(*μ*→3*γ*_0_).

Considering the appearance of new cavities in the Brillouin zone corners, one obtains from (4.6) and (4.9), if *T* → 0:4.14
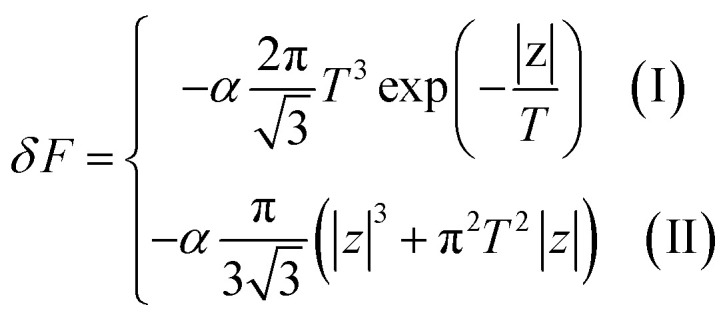
where |*z*| = *η*_0_|*S* − *S*_c_|, *η*_0_ = *η*(*μ*→0).

### 4.2. The specific heat of the graphene monolayer at the Lifshitz topological transition

The specific heat of graphene is stored in the lattice vibrations (phonons) and the free conduction electrons of graphene, *C* = *C*_ph_ + *C*_e_. However, phonons dominate the specific heat of graphene^58^ at all practical temperatures (>1 K),^59,60,*and*61,62^ and the phonon specific heat increases with temperature.^62^ The electron specific heat *C*_e_ of monolayer graphene (MG) is given by the following relations:^62^4.15
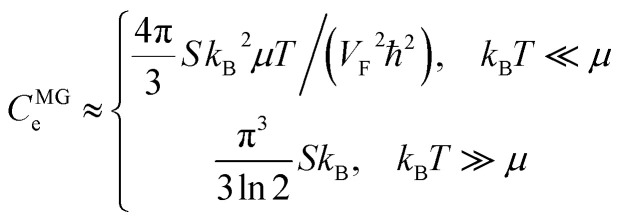
where *S* is the sample area and *k*_B_ is the Boltzmann constant.

We can explore the electron-specific heat peculiarities of monolayer graphene at the LTT based on expressions of the change of the Helmholtz free energy by eqn (4.12), (4.13), and (4.14).

In the case of the hyperbolic changes of the Fermi lines at the saddle point M, one obtains from (4.12), if *T* → 0:4.16
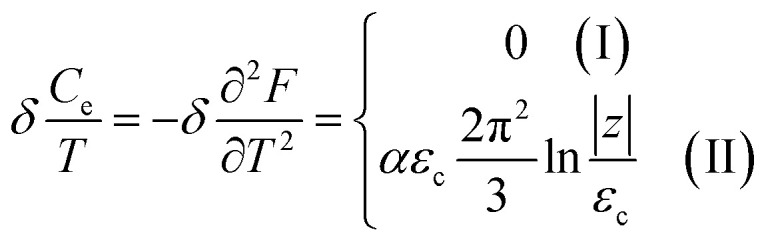
where *z* = *η*_*γ*_0__|*S* − *S*_c_|.

When the electronic cavity disappears in the Brillouin zone centre Γ, one computes from (4.13) at *T* → 0:4.17
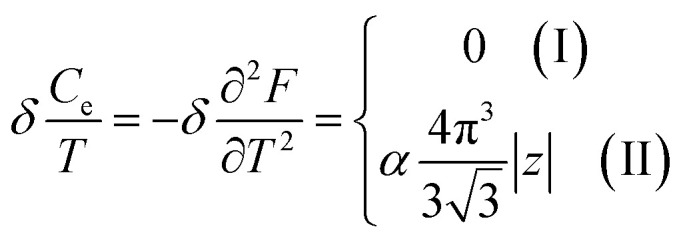


Considering the appearance of new cavities in the Brillouin zone corners K, we obtain from (4.14), if *T* → 0:4.18
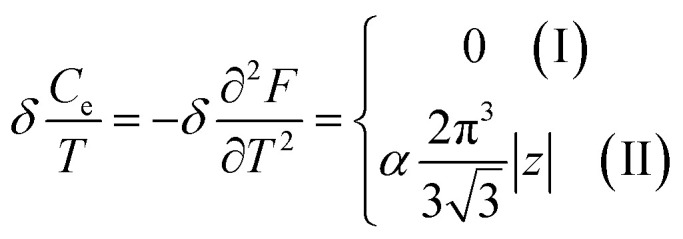
where |*z*| = *η*_0_|*S* − *S*_c_|, *η*_0_= *η*(*μ* → 0).

**Fig. 5:**
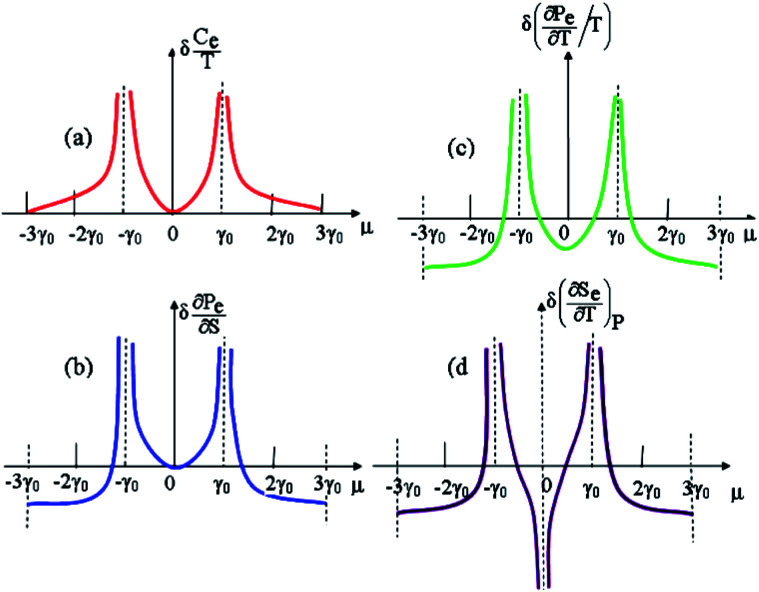
Peculiarities of the electronic thermodynamic parameters of graphene at the Lifshitz topological transitions at the points M (chemical potential *μ* = *γ*_0_), Γ (chemical potential *μ* = 3*γ*_0_), and K (chemical potential *μ* = 0) of the first Brillouin zone: (a) the electronic specific heat 
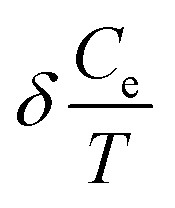
; (b) the electronic compressibility 
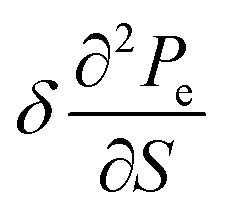
; (c) the electronic thermal coefficient of pressure 
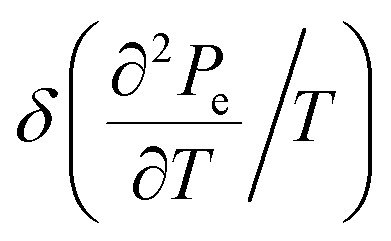
; and (d) the electronic thermal expansion coefficient 
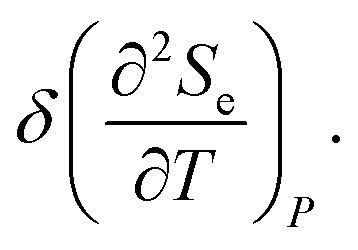

Thus, the formulae (4.16), (4.17) and (4.18) describe peculiarities of the specific heat in graphene at the LTT at the points M, Γ, and K of the first Brillouin zone, correspondingly (Fig. 5a).

The results for the specific heat (4.17) and (4.18) coincide with result (4.15) of the electron specific heat C^MG^_e_of the monolayer graphene (MG) obtained in paper.^61^ Indeed, substituting 
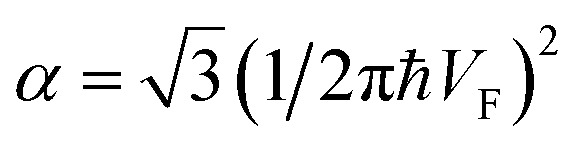
, and expressing *T* in Kelvins, one obtains from (4.17) and (4.18):

4.19 *C*_e_ ≈ *Sk*_B_^2^*μT*/(*V*_F_^2^*ħ*^2^), *k*_B_*T* ≪ |*z*| ≪ *μ*

Note that experimental observation of singularities (4.16) and (4.17) is possible under special conditions. The experimental value of the chemical potential *μ* of graphene does not exceed 1 eV,^63^ and the estimated value of the overlap integral *γ*_0_ is between 2 and 4 eV.^64–66^ Tuning of the chemical activity of graphene in a wide range could be achieved by the formation of a Moiré superstructure between the graphene and transition metal substrate.^67^ Thus, to observe the LTT at the graphene saddle point (where |*z*| = |*μ* − *γ*_0_| → 0), we must increase the chemical potential *μ* or decrease the overlap integral *γ*_0_ due to strain. Increasing *μ* can be realized by electron doping due to renormalization of the electron spectrum.^68^ It has been demonstrated in ref. 68 that the Coulomb interaction produces noticeable effects in increasing the graphene chemical potential *μ* at low temperature *T* ≪ *μ*, especially for high carrier concentrations of *n*_0_ > 10^11^ cm^−2^. The band structure of graphene has been determined under strain using density functional calculations.^69^ The *ab initio* band structure was then used to extract the best fit to the tight-binding hopping parameters used in a microscopic model of strained graphene.^70^ It was found that the hopping parameters may increase or decrease with increasing strain depending on the orientation of the applied stress. The fitted values were compared with an available parameterization for the dependence of the orbital overlap on the distance separating the two carbon atoms.

### 4.3. The compressibility of the graphene monolayer at the Lifshitz topological transition

The compressibility of graphene is an important object of study in condensed matter physics because it gives information about the intrinsic nature of the electron structure of graphene and its interactions with external fields.^71,72^

In case of the hyperbolic changes of the Fermi lines at the saddle point M, we obtain from (4.12) for the electron compressibility, if *T* → 0:4.20
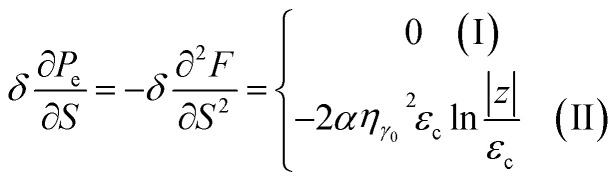
where *z* = *η*_*γ*_0__|*S* − *S*_c_|.

When the electronic cavity disappears in the Brillouin zone centre, one obtains from (4.13) for the electron compressibility at *T* → 0:4.21
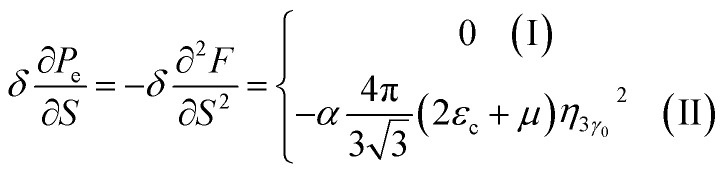
where *η*_3*γ*_0__= *η* (*μ* → 3*γ*_0_).

Considering the appearance of new cavities in the Brillouin zone corners, one obtains from (4.14) for the electron compressibility, if *T* → 0:4.22
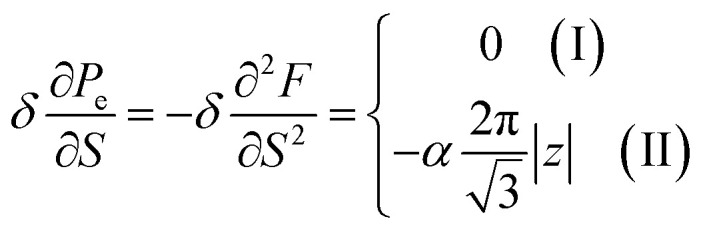
where |*z*| = *η*_0_|*S* − *S*_c_|, *η*_0_= *η*(*μ* → 0).

As a result, the formulae (4.20), (4.21) and (4.22) describe peculiarities of the compressibility of graphene at the LTT at the points M, Γ, and K of the first Brillouin zone, correspondingly (Fig. 5b).

### 4.4. The electron thermal coefficient of pressure of the graphene monolayer at the Lifshitz topological transition

In case of the hyperbolic changes of the Fermi lines at the saddle point A, we obtain the change of the electron thermal coefficient of pressure from (4.12), if *T* → 0:4.23
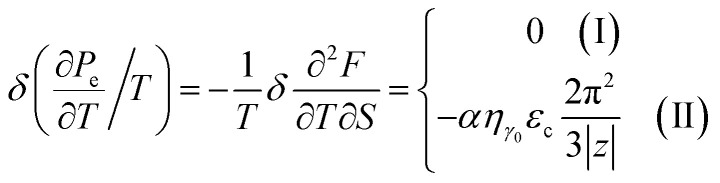
where *z* = *η*_*γ*_0__|*S* − *S*_c_|.

When the electronic cavity disappears in the Brillouin zone centre, one obtains from (4.13) for the electron thermal coefficient of pressure at *T* → 0:4.24
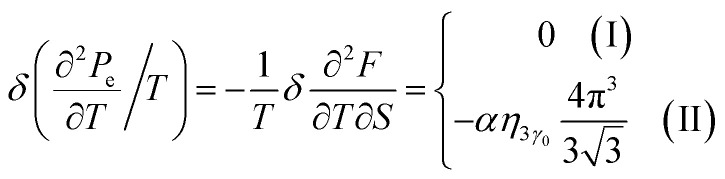
where *η*_3*γ*_0__= *η*(*μ* → 3*γ*_0_).

Considering the appearance of new cavities in the Brillouin zone corners, one obtains from (4.14) for the electron thermal coefficient of pressure, if *T* → 0:4.25
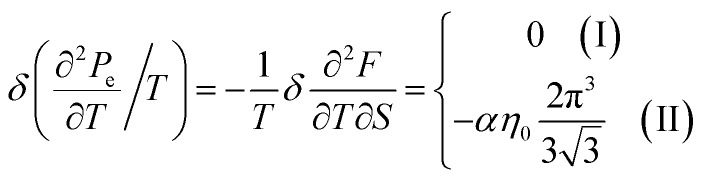
where *η*_0_ = *η*(*μ* → 0).

The formulae (4.23), (4.24) and (4.25) describe peculiarities of the electron thermal coefficient of pressure in graphene at the LTT at the points M, Γ, and K of the first Brillouin zone, correspondingly (Fig. 5c).

### 4.5. The electron thermal expansion coefficient of the graphene monolayer at the Lifshitz topological transition

The thermal properties of graphene have been investigated in recent years; in particular, its thermal expansion and heat conduction have been studied by various theoretical and experimental techniques in ref. 73 and 74. Some theoretical studies that have been carried out to study the thermodynamic properties of graphene (*e.g.*, specific heat and thermal expansion) are based on density-functional theory (DFT) calculations combined with a quantum quasi-harmonic approximation (QHA) for the vibrational modes.^73,74^ This is expected to yield reliable results at low temperature for the graphene lattice contribution to the thermal properties; however, it may be questioned with respect to the graphene electron part. The answer to the latter can give the Lifshitz topological transitions.

In the case of the hyperbolic changes of the Fermi lines at the saddle point M, we obtain the change of the electron thermal expansion coefficient from (4.23) and (4.20), if *T* → 0:4.26
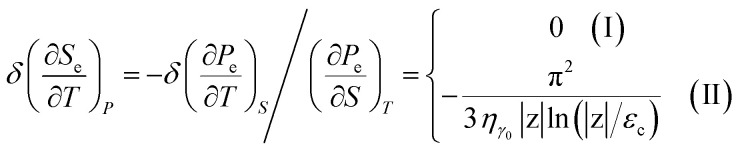
where *z* = *η*_*γ*_0__|*S* − *S*_c_|.

When the electronic cavity disappears in the Brillouin zone centre, one obtains from (4.24) and (4.21) for the electron thermal expansion coefficient, at *T* → 0:4.27
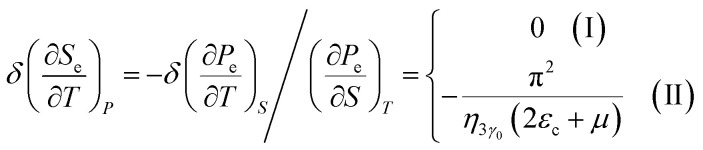
where *η*_3*γ*_0__= *η*(*μ* → 3*γ*_0_).

Considering the appearance of new cavities in the Brillouin zone corners, one obtains from (4.25) and (4.22) for the electron thermal expansion coefficient, if *T* → 0:4.28
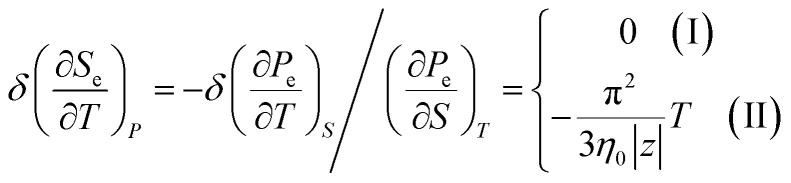
where *z* = *η*_0_|*S* − *S*_c_|.

It is interesting that the electron thermal expansion coefficient has a negative sign at *T* ≪ |*z*|, like the lattice thermal expansion coefficient of graphene (see ref. 72, 73, and 75).

The formulae (4.26), (4.27) and (4.28) describe the peculiarities of the electron thermal expansion coefficient in graphene at LTT at the points M, Γ, and K of the first Brillouin zone, correspondingly (Fig. 5d).

## 5 Conclusion and outlook

To summarize the results of the present paper, one can emphasize the following.

(i) Connection of the Lifshitz topological transition has been established with the van Hove singularities of the electron state density in graphene. There are three types of singularities of the density of the electron states in two dimensions. The point P_0_ (min) corresponds to the minimum in the energy spectrum. The point P_1_ (saddle) corresponds to the case where the Lifshitz topological transitions are realized by variation of the energy from *ε* < *ε*_c_ to ε >*ε*_c_. The point P_2_ (max) corresponds to the maximum in the energy spectrum.

(ii) Peculiarities of the Lifshitz topological transitions in graphene are described at the Brillouin zone centre Γ, at the zone corners K, in the vicinity of the Dirac points, and at the saddle point M. It is found that LTT can be realized in the centre Γ at the critical energy value *ε*_c_ = 3*γ*_0_, where the cavity of the isoenergetic lines disappears. The existence of the LTT is shown at the critical energy value of *ε*_c_ = 0, where six pockets of low energy excitations appear, one for each of the two inequivalent Dirac points K and K′.

The Lifshitz topological transition is realized in the saddle point M by variation of the energy from *ε* < *ε*_c_ to *ε* > *ε*_c_ and at the critical value of energy *ε*_c_ = *γ*_0_. It is shown that the Dirac fermion slows down in the vicinities of the points Γ and M, becoming not massless but massive, and the values of the fermion effective mass in the vicinity of these points are *m*^Γ^_eff_ ≈ 0.1*m*_e_, and *m*^M^_eff_ ≈ 0.1*m*_e_ (where *m*_e_ is the free electron mass).

(iii) The thermodynamic characteristics of graphene were investigated at the Lifshitz topological transitions. A general formulation of the thermodynamics at the LTT in graphene is given. The anomalies are found at the LTT of the electron specific heat *C*_e_, the electron compressibility 
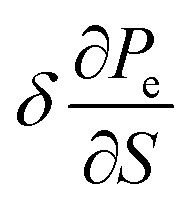
, the electron thermal coefficient of pressure 
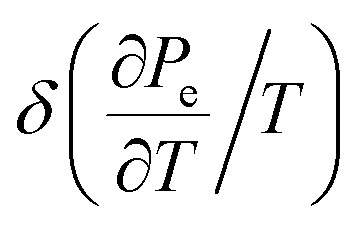
, and the electron thermal expansion coefficient 
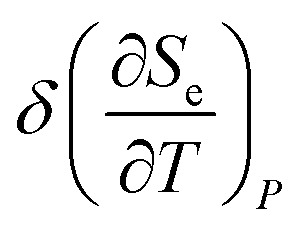
. The anomalies are described in terms of the Lifshitz parameter *z* = *μ* − *ε*_c_. The electron specific heat *C*_e_ diverges at the saddle point M as ln|*z*| and is proportional to |*z*| at points K and Γ of the first Brillouin zone. The electron compressibility 
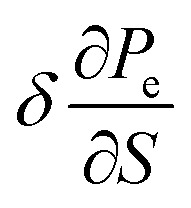
 diverges at the saddle point M as ln|*z*|, is proportional to |*z*| at points K, and takes a constant value at point Γ of the first Brillouin zone. The electron thermal coefficient of pressure 
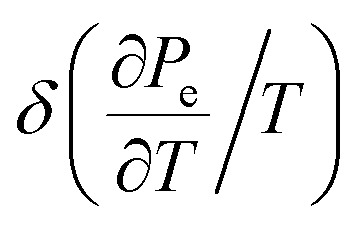
 diverges at the saddle point M as 1/|*z*|, and it becomes negative in the vicinities of points K and Γ of the first Brillouin zone. The electron thermal expansion coefficient 
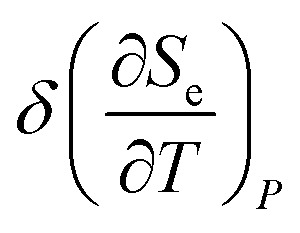
 diverges at the saddle point M as 1/(|*z*| ln |*z*|) becoming negative in the vicinity of point Γ, and it diverges as 1/|*z*| at point K of the first Brillouin zone.

One can conclude that all the thermodynamic parameters possess the strongest singularities in graphene at the LTT near the saddle points M. In 2D graphene, a saddle point M in the electronic band structure leads to a divergence in the density of states of the logarithmic-type van Hove singularities (VHS). This implies the possibility of experimental observation of the LTT by bringing the chemical potential μ and the VHS together. However, one cannot change the position of the VHS in the band structure. It is pointed out in section 4.2 that the accessible experimental value of the graphene chemical potential *μ* does not exceed 1 eV.^63^ Therefore, it is essential to tune μ through the VHS by chemical doping^76,77^ or by gating.^17,57,78–81^ In recent work,^82^ a simple technique of doping graphene by manipulating adsorbed impurities was reported, and a change in the electron mobility of 650% was observed. Also, it is worth paying experimental attention to the tuning of *μ* through the VHS by the following two methods. The first method is connected with deformation of the graphene monolayer to mimic twisted graphene. Rotation between two stacked graphene monolayers in twisted graphene^83^ can generate van Hove singularities, which can be brought arbitrarily close to the chemical potential *μ* by varying the angle of rotation.^84^ The second method consists of investigating the LTT in graphene under 3D high pressure.^84^ This opens exciting opportunities for inducing and exploring the Lifshitz topological transitions in graphene.

## Data accessibility

This work includes theoretical investigations.

## Authors' contributions

The paper has been written without any assistance, and I gave final approval for publication.

## Funding statement

The work has no source of funding.

## Ethics statement

This work did not involve any ethics statements.

## Conflicts of interest

I have no competing interests.

## Supplementary Material
